# Medical education in low- and middle-income countries must be innovated without neglecting its foundational principles

**DOI:** 10.3389/fmed.2026.1843757

**Published:** 2026-06-17

**Authors:** Sandro Vento

**Affiliations:** 1 Department of Clinical Disciplines, Manash Kozybayev North Kazakhstan University, Petropavlovsk, Kazakhstan; 2 Faculty of Medicine, University of Puthisastra, Phnom Penh, Cambodia

**Keywords:** artificial intelligence, curriculum, digital competency, low- and middle-income countries (LMICs), medical education, medical schools

## Introduction

In 2019, national health workforces lacked at least 6·4 million physicians, with the largest shortage in low-income settings (1). Medical schools in low- and middle-income countries (LMICs) need to be rethought and adapted to the needs of society and to a changing environment. This very difficult task must be accomplished if we wish to have good physicians and surgeons who will be able to diagnose and treat the population of those countries in the years to come. To reach this essential objective, public medical schools are of paramount importance, and their mission cannot be accomplished unless ministries of health are fully onboard and able to guarantee proper working conditions and career paths for newly graduated doctors. Adequate healthcare services and salaries of healthcare workers in the public sector are very important (2) to solve the issue of the export of medical doctors from LMICs that have invested in their training to high-income countries.

How can medical schools in LMICs (many of which currently have huge problems and deficiencies) (3) be up to the challenge?

## Purpose of medical schools in LMICs and role of ministries of health

Medical schools must graduate competent and compassionate physicians who are up to date with current trends in medicine. They must be able to help prevent diseases, to diagnose and treat sick people, to understand research data and its limits, and to mentor medical students and residents.

The ministries of health must have a leading role in planning the minimum number of medical graduates needed in the following 5–10 years, and the minimum and maximum number of students who should be enrolled in each medical school. Nowadays medical schools, following the example of Western ones, are obsessed with publicizing their admissions rankings, graduation rates, faculties' achievements and number of publications. These statistics are considered essential to foster accountability and improvement. However, as written by Muller (4) in his book about higher education, the Tyranny of Metrics, an “excellent method to increase graduation rates is to lower the standards for graduation” and demonstrating the accountability of a school through its excellent metric of performance; “the fact that no one has learned much, is beside the point.” This attitude must be avoided at all costs by any medical school, where the traditional reliance on experiential professional judgment must not be superseded by metric-driven assessments of comparative performance. Visibility must not be privileged over substance.

It is time for medical schools not to seek accreditations based on enormous compliance paperwork that may not reflect their reality but rather concentrate on making sure that their graduates are truly competent, based on competent and experienced faculty and on serious assessments. The purpose of any public medical schools in LMICs must be to graduate practicing doctors able to serve their own countries, not to prepare their graduates to do their residencies and then work in high-income countries. This is extremely important, as each country must consider what their peculiarities are and which aspects the medical curriculum must particularly focus on.

## Curriculum

The medical curriculum in any LMIC must meet the needs of the population of the country where the medical school is located. Planning is essential to avoid content overload, that leads to students' surface learning and rote memorization rather than proper understanding. To integrate basic with clinical sciences, to make students understand their relevance, to promote deep learning, to learn communication, collaboration, respect, leadership and to encourage self-directed learning are all ambitious goals of any curriculum in whatever medical school nowadays. However, this easy rhetoric must be abandoned to look at the difficult practice, i.e., whether a theoretically attractive curriculum is indeed able to produce competent doctors.

Two main issues must be considered when planning a curriculum. The first is today's concerning drive toward an unrealistic degree of “completeness” within an often-reduced timeframe. We live in an era of big data, predictive analytics, new medical devices, robotics and artificial intelligence (AI), where the art of medicine is considered obsolete, and physicians are being increasingly and erroneously moved away from direct patient contact. The second issue is the facilitation of transition from medical school to practice; in fact, most newly graduated physicians struggle more because they were not taught how to think clearly under real, stressful clinical pressure than because of lack of knowledge (5, 6).

In Western countries, assessments of learning can be divided into non-clinically-based, done in simulated clinical environments, and clinically-based (7). Six domains of competence are supposedly assessed: patient care, medical knowledge, professionalism, interpersonal and communication skills, practice-based learning and improvement, and systems-based practice (8).

In LMICs, we need to make sure that medical students know all the essentials; hence, the curriculum must promote clinical reasoning, observational skills (visual symptoms of illness or injury), critical thinking, and must provide sufficient self-study time. Surgical, medical, obstetrical procedures and ultrasound need to be learned and practiced by any individual student. The threshold to pass any assessments must not be low, and if students fail exams, they should re-take them. In addition, in an era where cheating is increasingly common (9–12), the role of oral exams, largely used in the past, must not be overlooked, if we want to make sure that both students' knowledge and critical thinking are assessed (13–16). The rhetoric of the risk that oral exams are influenced by lecturers' personal knowledge of the students and by their subjective opinion must be abandoned, as structured oral examination has shown high levels of validity and reliability (17). Without serious assessment of student learning, based also on oral exams, graduation cannot serve as evidence of learning.

Equity in healthcare must be learned and practiced. Students should help to provide care for vulnerable people (with well-defined competencies), and any public medical school should be committed to improving performance at a certain level of care (e.g., primary care) in a poor district/area of a city, or in villages.

The curriculum must be organized in such a way that research is understood, and students are able to write during their clinical years (under supervision) case reports to be submitted for publication; this writing would reinforce the notion that reaching a reasonably sure diagnosis requires appropriate investigation.

Finally, it must be considered that internship is an essential and very important training period (18). In LMICs, it should not be an extra 1 or 2 years after graduation but should rather be incorporated in the curriculum for medical schools to be able to control how well internship is done and whether supervision is present and appropriate. Too often those who do their internship after graduation to be allowed to practice independently are regarded in many instances as a much-needed extra pair of hands in a disorganized environment rather than doctors under probation still requiring supervision and training.

## Faculty balance between teaching, clinical commitment and research

Public medical schools should recruit the brightest clinicians in their countries and very good doctors from other countries. To succeed in this, meritocracy must be real and not just formal in both enrolment and subsequent promotion decisions.

Nowadays stakeholders' demand for measurable outcomes (e.g., graduate employability, number of publications and of their citations, presumed teaching quality, financial efficiency) has led to data-driven management, KPIs, and outcome-based funding of universities, similarly to corporate governance. Collegial governance has been replaced by managerialism and faculty autonomy is increasingly limited. With medical schools' reputation influenced by rankings in national and international league tables, the requirement for high numbers of largely irrelevant publications has led to a discouragement of long-term or high-risk projects, and to favoring safe, fashionable, short-term ones (19). Time to prepare for teaching and to teach has progressively decreased, with negative consequences on the development of students able to reflect and think critically. It is also dangerous recruiting or promoting professors with very limited clinical experience who got their titles based on number (rather than relevance) of publications and not on teaching and patient care.

LMICs must invest seriously in medical schools and in faculty quality. Teaching must be highly respected, well paid and well-supported. The relative importance of clinical commitment, teaching and research to recruitment and career progression (currently often unbalanced toward “research” consisting of publications) must be modified. As for research, a medical school in LMIC must not chase publications but try, if not to make new, significant discoveries, at least to contribute and find solutions to medical issues relevant to its own country and population.

Finally, education in a medical school must be at a university level, i.e., must integrate teaching with clinical experience, reflection and a critical understanding of recent findings. Teaching cannot be delivered by anyone in any subject. Permanent academic staff is therefore essential and must be commensurate with the student numbers; hiring inexperienced staff on short-term contracts can only eventually be a temporary solution. Having faculty with insufficient or no direct clinical and/or research experience in the taught subject/field who ask AI to make slides and read them, makes a bad medical school.

## Digital competencies and AI

The issue of AI is extremely important in nowadays medical education. Overreliance on it, with acceptance of AI-generated recommendations without question, affects the students' critical cognitive capabilities, including decision-making, critical thinking, and analytical reasoning (20). The curriculum must allow students to develop self-awareness of AI usage and help prevent excessive dependence on it and on mobile technology (21); critical thinking must be promoted and passive reliance on AI tools must be strongly discouraged. Hence, the contribution of psychologists and technology experts to curriculum design is essential.

AI has completely changed the process of intellectual maturity forming through engagement with difficulty, cohering and repetition that progressively improve judgment; automatic responses to questions and automatically summarized literature have made the labor of cognition, that previously required hours of reading and revising, disappear (22). The risk of surface-level synthesis and cognitive dependency is very high, whereas learning should be an active conversation between machine output and human understanding.

To start using AI as rapidly and largely as possible in medical schools and/or in healthcare is a mistake; students and doctors should rather stay up to date on AI and thoughtfully introduce and apply it.

An evidence-informed and consensus-guided framework can help in enabling medical schools to better prepare their future physicians for the digital transformation in health care (23). Obviously, medical schools in LMICs should judiciously adapt this framework to their needs and resources.

## Students need to be seriously selected

We live in an era of great difficulty for medical doctors worldwide. Seeing patients dying and then being blamed for it, getting litigation threats from patients and their relatives, being publicly criticized as “killers” on social media, newspapers and televisions lead good and experienced doctors to leave the profession. Young doctors learn not to take high-risk cases, defensive medicine increases (24), and the whole medical environment becomes unattractive. It is therefore essential to select students very carefully through the right selection process (25, 26). Importantly, enrolling in a medical school must not mean a guarantee of success or of getting a medical degree without being competent. The shortage of doctors in any country must not translate into graduating any medical student, even if unprepared and unfit for the profession (27).

## Conclusions

Public medical schools need bold and innovative leaders who steer their institutions toward genuine scholarship and are conscious of the huge importance for any LMIC of having good and competent doctors. Being innovative implies not only knowing and utilizing new developments, but also not being afraid to take initiatives against mainstream and not passively following current Western ideas about how a medical school must operate and about how teaching and assessments (28, 29) must be done. Faculty must not be expected to take huge extra lecture loads, teach multiple subjects outside their area of specialization, have assigned lengthy accreditation files, or have heavy non-academic work; they must be academics, not multitasking administrators. In fact, inadequate salaries and high non-academic workload cause demotivation, rote teaching, poor and non-innovative research. It is obviously important that each public medical school adapts to the culture of the country where it is located; however, this adaptation must not compromise the quality, skills and competence of the newly graduated doctors. Finally, it is very important not to focus heavily on internet visibility, with dedicated management of Instagram reels, LinkedIn posts, Facebook pages, and YouTube content showcasing all kinds of events and supposed achievements. Faculty and students must concentrate on teaching and learning rather than coordinating likes, shares, and engagement. The reputation of a medical school must be based on the validity of the physicians that come out of it, not on perception in reputation surveys for all kinds of rankings.
